# Compact Combustion Mechanisms of Typical *n*-Alkanes Developed by the Minimized Reaction Network Method

**DOI:** 10.3390/molecules28237695

**Published:** 2023-11-21

**Authors:** Jiangtao Shentu, Yanrong Lu, Yiwei Li, Juanqin Li, Yebing Mao, Xiangyuan Li

**Affiliations:** 1 School of Chemical Engineering, Sichuan University, Chengdu 610065, China; 2 Engineering Research Center of Combustion and Cooling for Aerospace Power, Ministry of Education, Sichuan University, Chengdu 610065, China; 3 School of Mechanical Engineering, Sichuan University, Chengdu 610065, China

**Keywords:** combustion mechanisms, minimized reaction network method, chemical resolution, reversible reactions, *n*-alkanes

## Abstract

The existing combustion kinetic modeling method which aims at developing phenomenological combustion mechanisms characterized by multiple reactions confronts several challenges, including the conflicts between computing resources and mechanism scales during numerical simulation, etc. In order to address these issues, the minimized reaction network method for complex combustion system modeling based on the principle of simultaneous chemical equilibrium is proposed, which is aimed to develop combustion mechanisms with minimal reaction steps under a limited number of species. The concept of mechanism resolution is proposed in this method, and the reaction network with minimal reaction steps under a given mechanism resolution is constructed so that the scale of mechanisms is compressed greatly. Meanwhile, distinguishing from other mechanisms, the reversible form of elementary reactions is adopted and the classical two-parameter (*A*, *E_a_*) Arrhenius equation fits the rate constants. Typical *n*-alkanes including *n*-butane, *n*-heptane, *n*-octane, *n*-decane, *n*-dodecane and *n*-hexadecane were taken as examples to indicate the development process of mechanisms and systematic kinetic validations were carried out. Results show that this method leads to very compact mechanisms with satisfactory accuracy, and it eliminates the process of mechanism reduction and is beneficial for mechanism optimization. This method and the derived kinetic mechanisms are hoped to contribute to combustion engineering applications.

## 1. Introduction

Due to the complexity of the combustion reaction process and its engineering application background in national defense and the national economy, combustion research has become an important interdisciplinary field [1,2]. Combustion is a chemical reaction process accompanied with luminescence and heating, in which active intermediate species play an important role. The combustion process generally involves the complex coupling of chemical reactions and fluid flow, thereby exhibiting various combustion characteristics. The micromechanisms are the basis of understanding the characteristics of combustion, while the combustion kinetics builds a bridge between the micromechanisms and the macrophenomena of combustion. Kinetic simulation of the combustion reaction process mainly includes two methods: one is phenomenological simulation based on lists of elementary reactions and another is molecular dynamics simulation based on a reactive force field (ReaxFF) [3].

In combustion chemistry, the kinetic process is generally described by kinetic mechanisms characterized by elementary reactions on the basis of the law of mass action. A common detailed mechanism is usually developed in the hierarchical manner [4,5]. The development of kinetic mechanisms is generally treated differently according to the carbon content of fuel molecules, which are mainly divided into small-molecule core mechanisms and macromolecular mechanisms. The core kinetic mechanisms mainly include H/O chemistry and C_1_-C_4_ chemistry, and some relatively mature mechanisms have been developed internationally [6,7]. For macromolecular fuels, the detailed combustion mechanisms are generally formed based on the core mechanisms. The scale of detailed combustion mechanisms increases exponentially with the increase in the number of carbon atoms of fuels. At present, the development of detailed combustion mechanisms of large hydrocarbon fuels generally depends on the automatic generation of computer programs [8,9].

Currently, there are relatively comprehensive understandings of the combustion process of hydrocarbon fuels, especially the common combustion reaction types and laws. A lot of progress has been achieved on the mechanism development of hydrocarbon fuels in recent years [4,10], which enriches the combustion reaction kinetic theories greatly. The combustion kinetic mechanisms of common single-component and real fuels, such as gasoline, aviation kerosene, diesel and biomass fuels, have been developed extensively, as well as the generation of soot [11,12] and -NO_x_ [13,14]. The development of mechanisms provides support for high-fidelity numerical simulation of engine combustion chambers, however, there still exist many problems needing to be solved [1].

Due to the high complexity of the reaction process, the cognitive depth of combustion is still not sufficient. In recent years, the development of quantum chemistry and computing power has promoted detailed reaction network research and the accurate calculation of reaction parameters. However, the diversity of molecular structure and the complexity of the combustion process determine the long-term nature of research on combustion kinetic mechanisms. It is still a severe challenge to develop the combustion kinetic mechanisms on the complete and reliable quantum chemical potential energy surface to achieve the numerical simulation. In addition, it is difficult to conduct experimental methods to investigate kinetic processes in combustion owing to the complex intermediate radicals and a great number of elementary reactions. As a result, many reaction pathways and kinetic parameters are proposed based on analogies or empirical estimates, which limits the accuracy of kinetic models.

In order to improve the predictive ability of kinetic models, researchers attempt to expand the mechanism size so as to include all possible reaction pathways in the detailed mechanism [15,16], leading to the increasing mechanism scale. However, due to the limited understanding of complex combustion processes such as free radicals and excited states, the way of trying to exhaust the reaction pathways is unrealistic. On the other hand, the more detailed mechanisms are undoubtedly of great significance to help understand the chemical reaction details of the combustion process, but it also leads to difficulties in applications of engineering numerical simulation directly. In terms of actual applications, the description of flow field structure in the internal flow channel of engines through numerical simulation of turbulent combustion plays a particularly important role in engine design and combustion organization. On the basis of the developed mechanisms, numerical simulation is able to obtain the change in species concentration, temperature and pressure under certain initial and boundary conditions [17,18]. As a matter of fact, the most important purpose of combustion mechanism development is still the numerical calculation of the flow field coupled with reactions. Due to the limitation of calculation capacity, the simulation of the combustion flow field requires the kinetic mechanisms to be as concise as possible, so the contradiction between the increasing scale of detailed kinetic mechanisms and the target of practical applications grows much more serious, which deviates from the original intention of kinetic mechanism development. There have been significant studies conducted on decreasing the scale of mechanisms through mechanism reduction and other methods [19,20,21], but it requires very complex processes to achieve the goals, especially when the detailed mechanisms are complicated.

Another characteristic of the current mechanisms is that many irreversible steps are adopted instead of reversible reactions [22,23]. An irreversible step means that when the reactants and products reach equilibrium, theoretically, it will still push forward the thermodynamic antispontaneous process, which obviously does not conform to the basic principle of thermodynamics.

In addition, the kinetic rate constants of reactions currently included in the mechanisms generally adopt the analytical formula of three parameters (*A*, *n*, *E*), corresponding to the modified Arrhenius equation:

(1) $$k=AT^{n}e^{-\frac{E}{RT}}$$

Herein, *A* is the pre-exponential factor, *T* is the temperature, *n* is the temperature index, *E* is the pseudo energy dimension and *R* is the universal gas constant. However, the parameters (*A*, *n*, *E*) fitted by the same set of rate constants (*k~T*) are not unique [24], which may depend on the initial fitting value of *A*, *n*, *E* given in the process of fitting iterations. Furthermore, it is difficult to visually judge the rationality of parameters (*A*, *n*, *E*) according to the reaction type in engineering applications.

A reliable combustion mechanism represented by lists of reactions needs to solve two major problems: one is the reasonable complex reaction network, and the other is the validity of kinetic parameters, thermodynamic parameters and transport parameters. Multiple sets of mechanisms for the same fuel have been developed by various authors due to the lack of unified rules as well as different targets. Owing to the differences in reaction types, reaction pathways and kinetic parameters of different mechanisms, it is difficult to compare, analyze and judge the reliability of different combustion mechanisms. There is a lack of consensus on how to obtain a comprehensive, unified and predictive model of fuel combustion [25].

In this work, the minimized reaction network method based on the principle of simultaneous chemical equilibrium for combustion mechanisms is further proposed and compact mechanisms of typical *n*-alkanes were developed through this method.

## 2. Results and Discussion of the Developed Mechanisms for *n*-Alkanes

Based on the proposed minimized reaction network method, compact kinetic mechanisms for *n*-butane, *n*-heptane, *n*-octane, *n*-decane, *n*-dodecane and *n*-hexadecane were developed in this work. The current version of mechanisms can be accessed in the Supplementary Materials.

### 2.1. Reaction Mechanisms for Typical n-Alkanes

As commonly accepted, the extended mechanisms of larger components are directly developed on the basis of the H_2_/C_1_-C_3_ mechanism developed previously [8,26,27]. In the actual construction process, the mechanism of the single-component fuel is developed individually, and the scales for typical *n*-alkanes in this work are more generalized as mentioned before. Under the condition of specific species, the cracking network of fuel molecules is constructed in the meantime, which ensures that the main small-molecule products can be generated from large molecules when the fuel only undergoes cracking reactions. This is another different characteristic compared to the reduced mechanisms.

**Figure 1:**
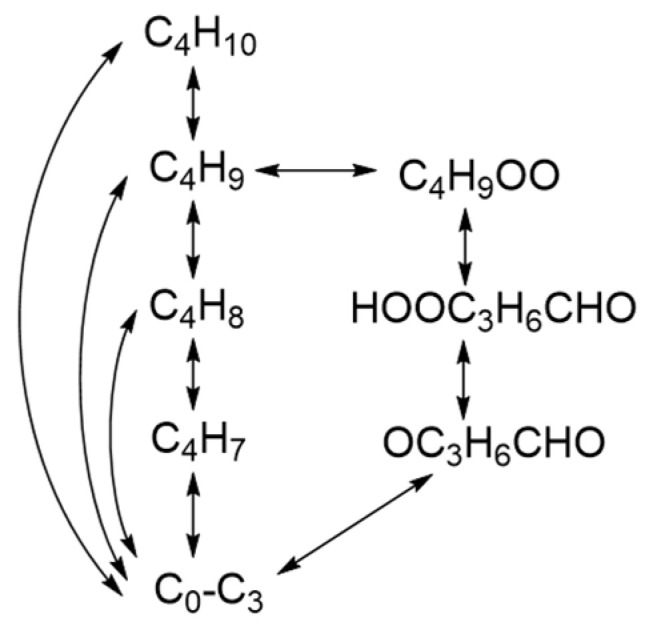
The main reaction network for the *n*-butane mechanism developed in this work.

The main reaction network for the combustion mechanism of *n*-butane developed in this work is shown in Figure 1, in which C_4_H_10_ rapidly proceeds to the H_2_/C_1_-C_3_ mechanism through the designed high-temperature and low-temperature paths. The extended C_4_ reactions with their kinetic parameters are presented in Table 1. Only the species of C_4_H_9_, C_4_H_8_ and C_4_H_7_ are considered in the high-temperature paths. The C_4_H_10_ is able to generate C_2_H_5_ and C_4_H_9_ through pyrolysis reactions, (R1) C_4_H_10_(+M)<=>2C_2_H_5_(+M) and (R2) C_4_H_10_<=>C_4_H_9_+H, while C_4_H_9_ can also be produced through oxidation reactions, (R3) and (R4). There are several cracking reactions of C_4_H_9_ as seen in (R5)–(R11), by which the production of sufficient small-molecule products from the pyrolysis process of macromolecular fuels is ensured. The C_4_H_8_ and C_4_H_7_ formed by C_4_H_9_ also quickly produce H_2_/C_1_-C_3_ small-molecule species through simple reaction paths. The low-temperature reaction path is mainly formed by the peroxyl C_4_H_9_OO generated by reaction (R12) and the subsequent low-temperature channels (R13)–(R15). Significantly, this low-temperature path is still treated in a reversible manner. In contrast, small-molecule products are rapidly generated from peroxyl radicals through non-elementary irreversible reactions adopted in certain mechanisms [6,7]. The mechanism for *n*-butane developed in this work includes 41 species and 83 reactions in total.

**Figure 2:**
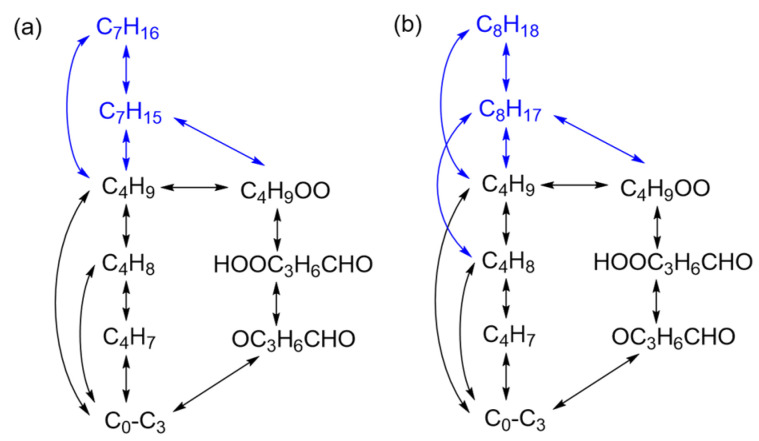
The main reaction network for the *n*-heptane and *n*-octane mechanism developed in this work: (**a**) *n*-heptane; (**b**) *n*-octane. (The blue part is newly added on the basis of the previous mechanisms.)

The combustion mechanism for *n*-heptane is developed on the basis of the H_2_/C_1_-C_4_ mechanism as the *n*-butane mechanism has been acquired and partial species can be shared. The main reaction network of *n*-heptane in this work is shown in Figure 2a and the added reactions above C_4_ with their kinetic parameters are presented in Table 2. The blue part in Figure 2a indicates new species and reaction channels in which only C_7_H_16_ and C_7_H_15_ are considered. C_7_H_15_ and C_4_H_9_ can be generated from C_7_H_16_ through cracking reactions (R21)–(R22) while C_7_H_15_ can also be produced through hydrogen abstraction reactions (R23)–(R25) as well. The consuming steps for C_7_H_15_ are designed by pyrolysis reaction (R26) as well as the low-temperature reaction channel through reaction (R27). In order to achieve the negative temperature coefficient (NTC) behavior and further reduce the number of species as well as compress the reaction network as much as possible, the low-temperature reaction channel of C_4_H_9_ is shared for all larger *n*-alkanes in the network construction. Additionally, the ignition delay times under low-temperature conditions is guaranteed to have a certain NTC behavior by adjusting the rate constant of the newly added reactions. The mechanism for *n*-heptane developed in this work includes 42 species and 86 reactions.

As for *n*-octane, a reaction network similar to the *n*-heptane mechanism is adopted as shown in Figure 2b and Table 3. Similarly, only two species, C_8_H_18_ and C_8_H_17_, are added on the basis of the H_2_/C_1_-C_4_ mechanism. The main reaction types are almost the same as those of the *n*-heptane mechanism. The slight difference is that C_8_H_17_ directly generates another C_4_ species of C_4_H_8_ which firstly appeared in the *n*-butane mechanism, yet no species are newly raised due to the existence of the C_4_H_9_ → C_4_H_8_ pathway. The mechanism for *n*-octane includes 42 species and 86 reactions in total.

**Figure 3:**
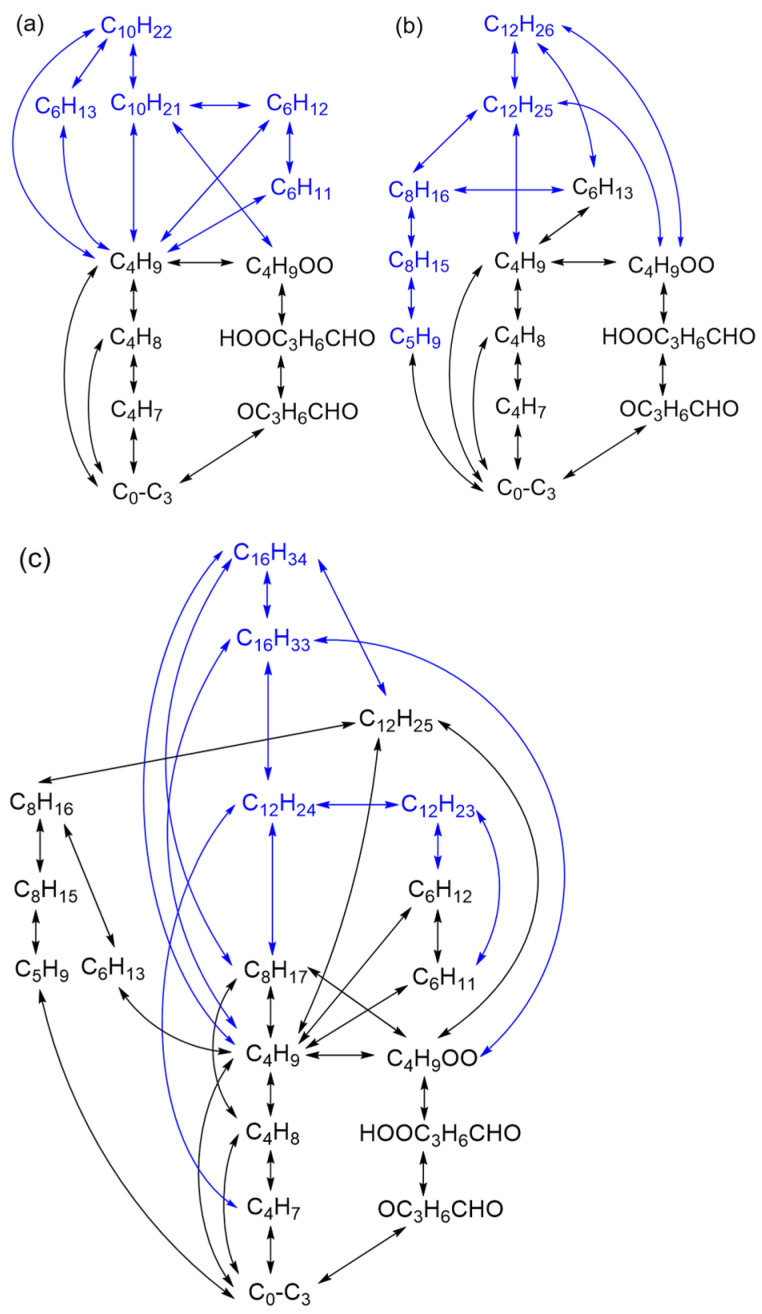
The main reaction paths for the *n*-decane, *n*-dodecane and *n*-hexadecane mechanisms developed in this work: (**a**) *n*-decane; (**b**) *n*-dodecane; (**c**) *n*-hexadecane. (The blue part is newly added on the basis of the previous mechanisms.)

For the combustion mechanism of *n*-decane, the main network is shown in Figure 3a in which the blue part emphasizes the added species and reaction channels. The newly added reactions with their kinetic parameters are presented in Table 4. C_10_H_21_, C_6_H_13_ and C_4_H_9_ are designed to be generated through the pyrolysis of C_10_H_22_. Afterwards, C_6_H_12_ can be produced through the decomposition of C_10_H_21_ and another consumption channel of C_10_H_21_ depends on the low-temperature pathway (R41) to generate C_4_H_9_OO and C_6_H_12_ in the meantime. The subsequent reactions are carried out by C_6_H_13_ and C_6_H_12_ via their cracking and oxidation steps as shown in reactions (R42)–(R46). The behavior of NTC comes from the production of C_4_H_9_OO as well as its subsequent reactions. The mechanism for *n*-decane includes 45 species and 91 reactions altogether.

For the combustion mechanism of *n*-dodecane, C_12_H_26_, C_12_H_25_, C_8_H_16_, C_8_H_15_ and C_5_H_9_ are additional species on the basis of the previous mechanism. The specific main reaction network is shown in Figure 3b and the extra reactions with their kinetic parameters are shown in Table 5. C_6_H_13_ can be directly obtained by C_12_H_26_ through a cracking channel, meanwhile C_12_H_25_ generated by C_12_H_26_ entering the existing C_10_H_21_ reaction pathway through reaction (R52). Furthermore, C_8_H_16_ and subsequent reactions are generated from the low-temperature step (R53) in which C_4_H_9_OO is produced by C_12_H_25_. The mechanism for *n*-dodecane includes 46 species and 91 reactions in total.

For the combustion mechanism of *n*-hexadecane, the main reaction network and newly added reactions with kinetic parameters are presented in Figure 3c and Table 6 correspondingly. C_16_H_34_, C_16_H_33_, C_12_H_24_ and C_12_H_23_ are given priority to be considered on the basis of the previous mechanism. C_16_H_34_ is consumed to form C_8_H_17_ or C_16_H_33_ by reactions (R58)–(R63). Subsequently, the consumption channels of C_16_H_33_ are decomposition to generate C_12_H_24_ and C_4_H_9_ as well as the low-temperature oxidation reaction (R65) to produce C_4_H_9_OO and C_12_H_24_. There are different consumption pathways for C_12_H_24_ which gradually enters the steps of small-molecule products. The mechanism for *n*-hexadecane includes 52 species and 104 reactions.

### 2.2. Mechanism Validations

In the process of reaction network construction and overall optimization of kinetic parameters, the macroindicators for fuel combustion were mainly validated focusing on the ignition delay time (IDT) and laminar flame speed (LFS). As the mechanisms developed in this work are not concentrated on the low-temperature part at present and low-temperature channels were treated with an extremely simplified process, the ignition delay times for developed mechanisms have only achieved a rough trend in the NTC behavior. The performance of developed mechanisms was compared with some experimental values reported in the literature. All the validations of kinetic simulation were completed in the Chemkin-Pro package [28].

#### 2.2.1. *n*-Butane

**Figure 4:**
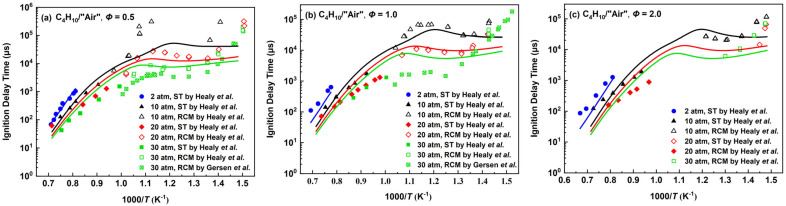
Ignition delay times for the *n*-butane mechanism compared with experimental data by Healy et al. [29] and Gersen et al. [30]. The four lines are the predictions at 2, 10, 20 and 30 atm, from top to bottom. (**a**) *Φ* = 0.5; (**b**) *Φ* = 1; (**c**) *Φ* = 2.

The simulation results of ignition delay times for the *n*-butane mechanism are shown in Figure 4, which are mainly compared with the experimental work conducted by Healy et al. [29] and Gersen et al. [30]. The pressure range of the working condition is 2–30 atm and the equivalence ratio range is 0.5–2.0.

When the equivalence ratios are 1.0 and 2.0 in the high-temperature range, the ignition delay times tend to be shorter than the experimental values with increasing temperature, which is because the performance of fuel cracking was taken into account in the development process and cracking reactions with faster parameters were adopted. When the equivalence ratio is 0.5, the simulation results of the *n*-butane mechanism are relatively consistent with the high-temperature experimental values compared with other equivalent ratios. As the temperature approaches the middle-temperature transition region of high temperature and low temperature, the simulation results of the *n*-butane mechanism are generally greater than the experimental values, which is a compromise effect to balance the effects of high and low temperature. In the mid-to-low-temperature region, the simplified low-temperature steps designed in this work can show an obvious NTC phenomenon and there exist certain deviations because of the small number of reactions. When the equivalence ratio is 0.5 and the pressure is 30 atm in Figure 4a, the experimental values conducted by Gersen et al. were completed in the “air” composed of O_2_/Ar and the ignition delay times are generally shorter than the results in the mixture of O_2_ and N_2_ obtained by Healy’s work in which the difference was discussed [29]. The *n*-butane mechanism can predict the experimental results on a trend in the wide temperature range and the deviations should be tolerable in the case of a small number of species and reactions.

**Figure 5:**
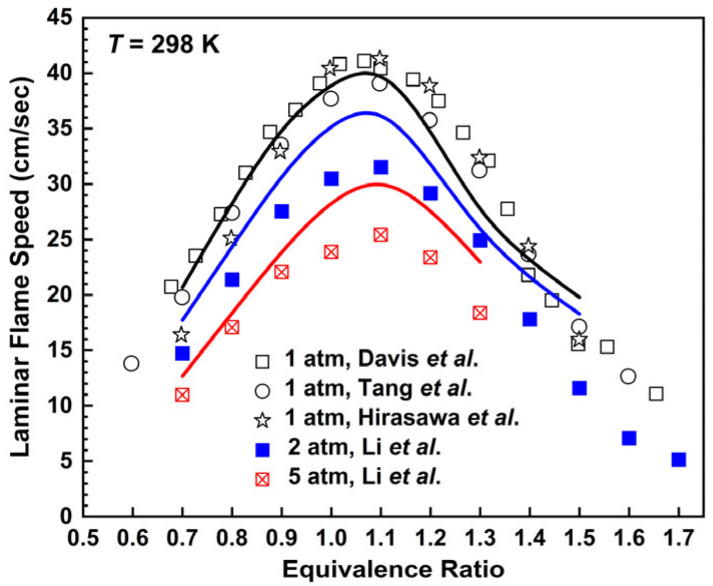
Laminar flame speeds for *n*-butane/air mixtures at 298 K. Lines are mechanism predictions at 1, 2 and 5 atm, from top to bottom. Symbols are experimental data by Davis et al. [31], Tang et al. [32], Hirasawa et al. [33] and Li et al. [34].

The results of laminar flame speeds are presented in Figure 5, in which the *n*-butane mechanism is compared with the experimental values at 298 K according to the work of others [31,32,33,34]. Under the working condition of 1 atm, the *n*-butane mechanism can predict the changing trend of laminar flame speeds affected by equivalence ratios properly, in which it is basically consistent with the experimental values at the equivalence ratio range of 0.7–1.5 and slightly slower than the experimental values in the equivalence ratio range of 1.2–1.4. Under the working condition of 2 atm, the *n*-butane mechanism is faster than the experimental values near the equivalence ratio around 1.1 and the maximum error is about 15%. When the equivalence ratios are over 1.3, the simulation values tend to be faster than the experimental values and similar results can also be obtained when the pressure is 1 atm. At 5 atm, there are similar conclusions for the *n*-butane mechanism as at 2 atm.

In general, the *n*-butane mechanism developed in this work can predict the ignition delay times and laminar flame speeds to a certain extent with fewer species and reactions.

#### 2.2.2. *n*-Heptane

**Figure 6:**
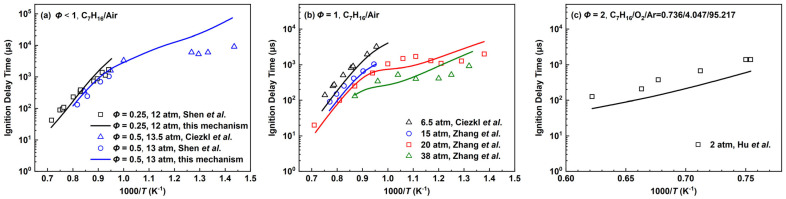
Ignition delay times for the *n*-heptane mechanism compared with experimental data by Zhang et al. [35], Ciezkl et al. [36], Shen et al. [37] and Hu et al. [38]. Lines are mechanism predictions, (**a**) 12 and 13 atm; (**b**) 6.5, 15, 20 and 38 atm; (**c**) 2 atm, from top to bottom.

The ignition delay times covering different equivalence ratios and pressures for the *n*-heptane mechanism are compared with experimental values given by Zhang et al. [35], Ciezkl et al. [36], Shen et al. [37] and Hu et al. [38]. Figure 6a includes some fuel-lean conditions, Figure 6b mainly shows the equivalence ratio of 1.0 covering pressures from 6.5 atm to 38 atm and Figure 6c is for the fuel-rich results.

At pressures below 15 atm, the reference experimental values only include high-temperature conditions, and the simulation results of the *n*-heptane mechanism are generally shorter and the difference becomes more obvious at higher temperatures. For the operating conditions including low-temperature experiments, the pressures are 20 atm and 38 atm, respectively. At *p* = 20 atm and *T* ≈ 1050–1200 K, the simulation results of the mechanism present a good agreement with the experimental data while the ignition tends to be shorter than the experimental values as the temperature rises. For the medium-to-low-temperature regions in which the NTC behavior is involved, the *n*-heptane mechanism can only present a broad trend. The C_7_H_15_ radicals are connected to the low-temperature path of C_4_ through reaction (R27), which mainly drives the IDTs to show a certain NTC behavior. Limited to the number of species and reactions, the deviations become more obvious as the temperature decreases.

For the high-temperature and lean-burn condition of *Φ* = 0.25 and *p* = 12 atm, there is a certain error between the line slope of the simulation results and experiment values, in which the simulated IDTs are longer than the experimental values when approaching the middle temperature about 1000 K. Meanwhile, the simulated IDTs are shorter than the experimental values at a higher temperature. At *Φ* = 0.5 and *p* = 13 atm, the simulated IDTs are slightly longer than the experimental values in the high-temperature range and the difference is more obvious at a lower temperature. When there are fuel-rich high-temperature conditions as shown in Figure 6c, the *n*-heptane mechanism predicts shorter IDTs at low pressure.

**Figure 7:**
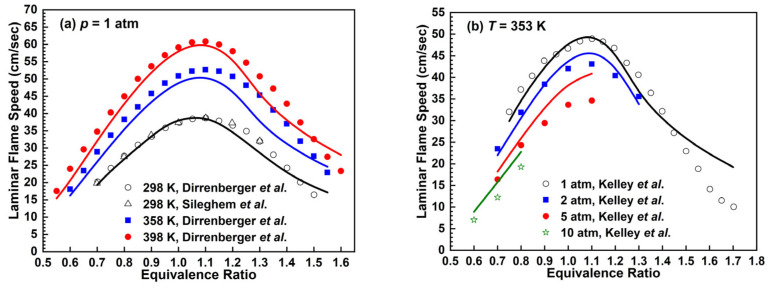
Laminar flame speeds for *n*-heptane/air mixtures. Lines are mechanism predictions, (**a**) 298, 358 and 298 K; (**b**) 1, 2, 5 and 10 atm, from top to bottom. Symbols are experimental data by Dirrenberger et al. [39], Sileghem et al. [40] and Kelly et al. [41].

The prediction results of laminar flame speeds in various conditions are presented in Figure 7, which are compared with the experimental results by Dirrenberger et al. [39], Sileghem et al. [40] and Kelley et al. [41]. At *p* = 1 atm and in fuel-lean conditions, the *n*-heptane mechanism is in good agreement with experimental data. With the increment of the equivalence ratio, the predicted laminar flame speeds are slower than the experimental values at an equivalence ratio of 1.1–1.4, while the laminar flame speeds tend to be faster than the experimental values when the equivalence ratio further increases. In Figure 7b, the *n*-heptane mechanism predicts faster results more obviously at *p* = 1 atm, *Φ* > 1.4 and *p* = 5 atm, *Φ* = 0.9–1.1.

In general, the results of ignition delay times and laminar flame speeds for the *n*-heptane mechanism can be basically accepted under different working conditions when only two species, C_7_H_16_ and C_7_H_15_, are added to the basic mechanism containing smaller species developed previously.

#### 2.2.3. *n*-Octane

**Figure 8:**
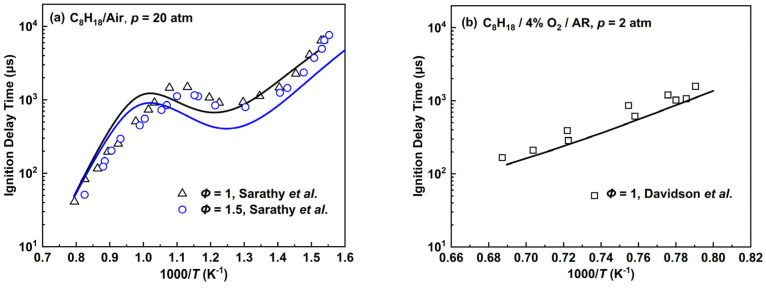
Ignition delay times for the *n*-octane mechanism compared with experimental data by Sarathy et al. [42] and Davidson et al. [43]. Lines are mechanism predictions, (**a**) *p* = 20 atm, *Φ* = 1 and 1.5; (**b**) *p* = 2 atm, *Φ* = 1.

The predicted ignition delay times for the *n*-octane mechanism were verified under different conditions and compared with the experimental values as shown in Figure 8. Under the conditions of 20 atm and *Φ* = 1.0 in Figure 8a, the IDTs are basically consistent with the experimental data at a high temperature of about 1200 K. As the temperature approaches the middle zone, the IDTs are firstly longer than the experimental data and then become shorter than the reference values. Finally, there has been a convergence in the simulation and experimental values in the low-temperature range. Owing to similar reaction types and network to that of the *n*-heptane mechanism, the NTC behavior of the *n*-octane mechanism mainly comes from (R34) and its subsequent reactions. As a comparison, the predicted tendency of the *n*-octane mechanism at *Φ* = 1.5 is generally similar to the results at *Φ* = 1.0, except that it is relatively shorter than the experimental data at a lower temperature. However, the experimental values are virtually identical at different equivalence ratios, while the *n*-octane mechanism overpredicts this difference. At the lower pressure of 2 atm and an equivalence ratio of 1.0 in Figure 8b, the simulation results of the *n*-octane mechanism are marginally shorter than the experimental values. In general, the *n*-octane mechanism reflects the NTC behavior in the medium-temperature range to some extent and the trend of the IDTs for the *n*-octane mechanism can be predicted in a wide temperature range through two species and seven reactions added to the previously developed mechanisms.

**Figure 9:**
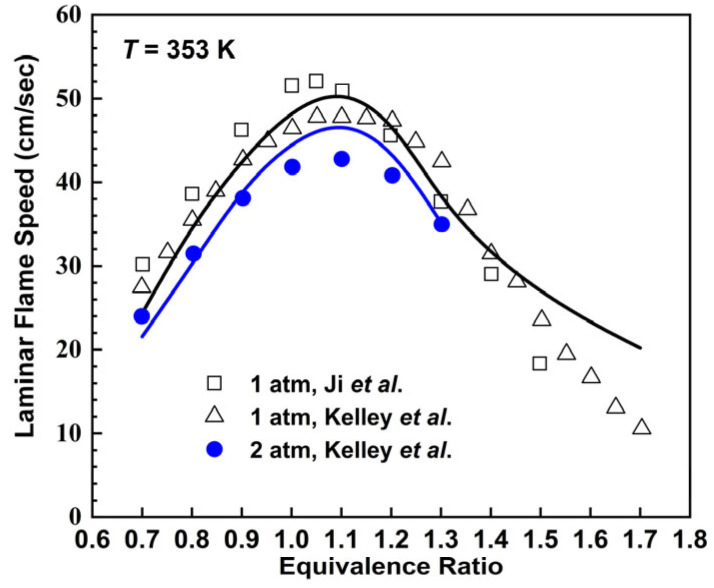
Laminar flame speeds for *n*-octane/air mixtures at 353 K. Lines are mechanism predictions at 1 and 5 atm, from top to bottom. Symbols are experimental data by Ji et al. [44] and Kelly et al. [41].

The laminar flame speeds for the *n*-octane mechanism varying with equivalence ratios at *T* = 353 K and *p* = 1, 2 atm are shown in Figure 9, which are compared with experimental values derived from the work of Ji et al. [44] and Kelley et al. [41]. At *p* = 1 atm, the *n*-octane mechanism is able to describe the changing trend of experimental laminar flame speeds when *Φ* < 1.4, and the *n*-octane mechanism tend to be faster than experimental values when *Φ* > 1.5. At *p* = 2 atm, there exist certain deviations between the predicted results and experimental values when *Φ* = 1.0–1.2.

#### 2.2.4. *n*-Decane

**Figure 10:**
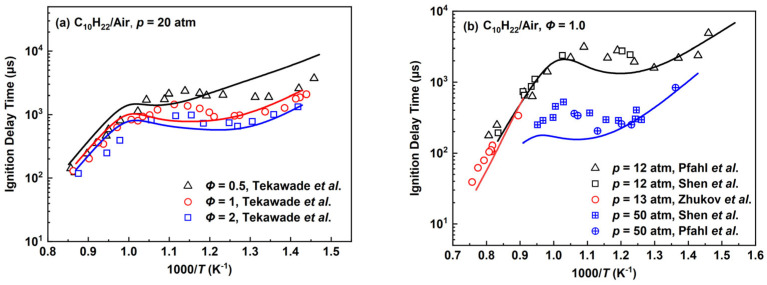
Ignition delay times for the *n*-decane mechanism compared with experimental data by Tekawade et al. [45], Pfahl et al. [46], Zhukov et al. [47] and Shen et al. [37]. Lines are mechanism predictions, (**a**) *Φ* = 0.5, 1 and 1.5; (**b**) 12, 13 and 50 atm, from top to bottom.

The verification results of ignition delay times for *n*-decane/air mixtures were found in different experimental conditions as illustrated in Figure 10, which mainly cover the temperature range of about 700–1300 K at pressures of 12–50 atm and equivalence ratios of 0.5–2.0. The *n*-C_10_H_22_ proceeds to the C_4_ mechanisms through C_10_H_21_, C_6_H_13_, C_6_H_12_ and C_6_H_11_ as shown in Figure 3a. The contribution in the NTC behavior mainly comes from C_4_H_9_OO generated by (R41) and its subsequent reactions, while C_4_H_9_ generated by (R35), (R42), (R45), (R46) and their subsequent reactions also has some influence.

The experimental values at 20 atm in Figure 10a are taken from the work by Tekawade et al. [45]. The predicted IDTs of the *n*-decane mechanism are longer than those of Tekawade et al.’s work in the high-temperature range to some extent, while the results of the *n*-decane mechanism are gradually shorter than the experimental values in the medium-temperature range. For the results in the low-temperature range, the IDTs of the *n*-decane mechanism are obviously longer compared with experimental data at the equivalence ratio of 0.5, while the predicted results are somewhat closer to the experimental data at the equivalence ratios of 1.0 and 2.0. The simulation results at lower pressures of 12, 13 atm and the equivalence ratio of 1.0 are shown in Figure 10b to compare with the experimental values by Pfahl et al. [46], Zhukov et al. [47] and Shen et al. [37]. The *n*-decane mechanism has shorter IDTs in the high temperature range and the NTC region. Similar results can be acquired as the pressure increases further to 50 atm, in which the relative errors of predicted IDTs in the mid-temperature region tend to be more obvious compared to *p* = 12 atm. As a whole, the *n*-decane mechanism generally predicts the variation trend of ignition delay times under different operating conditions.

**Figure 11:**
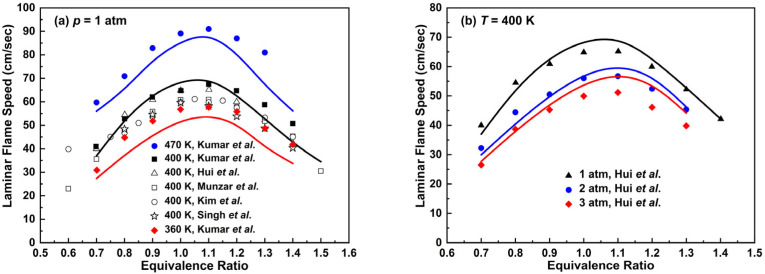
Laminar flame speeds for *n*-decane/air mixtures. Lines are mechanism predictions, (**a**) 470, 400 and 360 K; (**b**) 1, 2 and 3 atm, from top to bottom. Symbols are experimental data by Munzar et al. [48], Kim et al. [49], Hui et al. [50], Singh et al. [51] and Kumar et al. [52].

The comparison of laminar flame speed prediction for the *n*-decane mechanism with experimental data is presented in Figure 11. At *p* = 1 atm and *T* = 400 K, the *n*-decane mechanism is able to reproduce the trend of experimental laminar flame speeds along with the change in equivalent ratio to a good degree. It is worth noting that when the equivalence ratio is 0.6, there exists an obvious difference between the experimental data of Munzar et al. [48] and Kim et al. [49]. At *T* = 360 and 470 K, the *n*-decane mechanism underestimates laminar flame speeds compared to experimental data. In Figure 11b, the *n*-decane mechanism tends to overestimate laminar flame speeds as pressure and equivalence ratio increase.

#### 2.2.5. *n*-Dodecane

**Figure 12:**
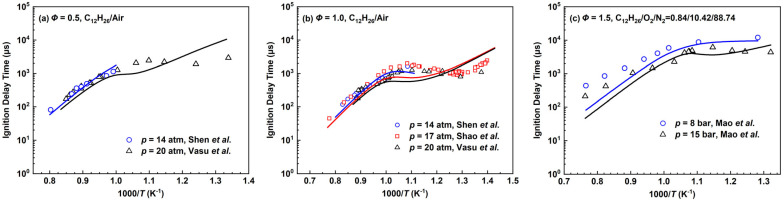
Ignition delay times for the *n*-dodecane mechanism compared with experimental data by Shen et al. [37], Shao et al. [53], Vasu et al. [54] and Mao et al. [55]. Lines are mechanism predictions, (**a**) 14 and 20 atm; (**b**) 14, 17 and 20 atm; (**c**) 8 and 15 bar, from top to bottom.

The results of IDTs for the *n*-dodecane combustion under different conditions are presented in Figure 12. For the high-temperature conditions at the pressure of 14 atm and equivalence ratios of 0.5 and 1.0, the simulation results are in good agreement with the experimental values by Shen et al. [37] in which the maximum temperature reaches up to about 1250 K. As for the simulation results in the mid-to-low-temperature region, the contribution of NTC behavior for the *n*-dodecane mechanism also mainly comes from C_4_H_9_OO generated by the fuel radical C_12_H_25_ through reaction (R53) and its subsequent reactions, while the remaining contributions come from C_4_H_9_OO generated by the intermediate species C_4_H_9_ and subsequent reaction pathways.

When the equivalence ratio is 1.0 and the pressure is 17 atm in Figure 12b, the IDTs are shorter than the experimental data at about 1300 K. The experimental temperature does not reach 1300 K at 20 atm, but similar results can be expected. As the temperature is further reduced to around 1000 K, the ignition delay times are still shorter than the experimental values to a certain extent. Notably, the experimental values by Shen et al. [37] at 14 atm are smaller than that by Shao et al. [53] at 17 atm. As the temperature becomes lower, the simulated IDTs become longer than the experimental data. Due to the small number of species and reactions involved in the low-temperature range, the current deviations become larger which is a disadvantage of the developed mechanisms in this work.

In Figure 12c, the *n*-dodecane mechanism is calculated in the fuel-rich conditions. The simulated results at a higher temperature are obviously shorter than experimental values, and the deviations become smaller as the temperature decreases.

**Figure 13:**
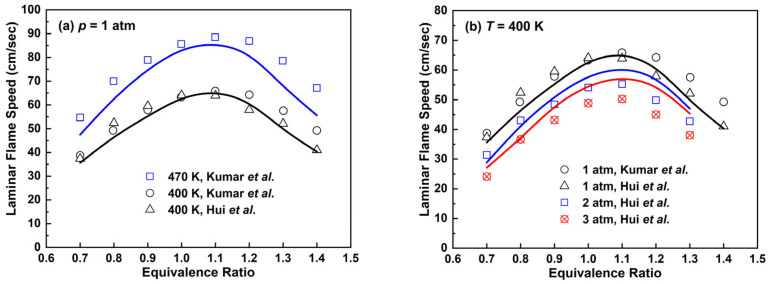
Laminar flame speeds for *n*-dodecane/air mixtures at 1 atm. Lines are mechanism predictions, (**a**) 470 and 400 K; (**b**) 1, 2 and 3 atm, from top to bottom. Symbols are experimental data by Kumar et al. [52] and Hui et al. [50].

The simulated laminar flame speeds of *n*-dodecane/air mixtures are presented in Figure 13 and the results were compared with the experimental work by Kumar et al. [52] and Hui et al. [50] under different conditions. At *p* = 1 atm and *T* = 400 K, the *n*-dodecane mechanism is basically consistent with experimental results. As the temperature increases to 470 K, the simulation results are slower than experimental values, especially under fuel-rich combustion conditions. There exists an error of about 20% when the equivalence ratio is 1.4. At *p* = 2 and 3 atm in Figure 13b, the simulation conclusions are similar to that of the *n*-decane mechanism in Figure 11b. Thus, further improvement is needed in the laminar flame speed prediction when pressure increases.

#### 2.2.6. *n*-Hexadecane

**Figure 14:**
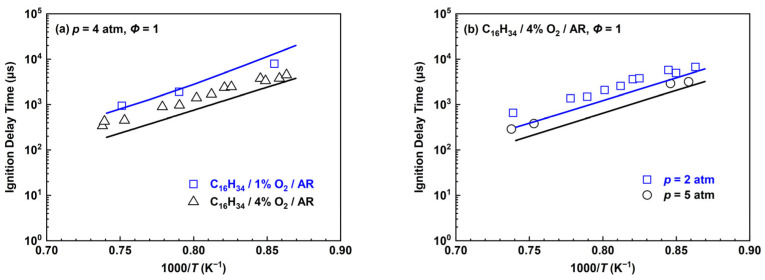
Ignition delay times for the *n*-hexadecane mechanism compared with experimental data by Haylett et al. [56]. Lines are mechanism predictions, (**a**) 1% O_2_ and 4% O_2_; (**b**) 2 and 5 atm, from top to bottom.

The experimental IDTs of the *n*-hexadecane mechanism in Figure 14 were found by Haylett et al. [56] in high-temperature conditions in which the equivalence ratio is 1.0 and the pressure range is 2–5 atm, respectively. When the pressure is 4 atm and the content of oxygen is 4%, the simulation line of the *n*-hexadecane mechanism is slightly lower than the experimental values. Similar situations exist in the work of Haylett et al. in which the mechanism developed by Westbrook et al. [15] was used. In addition, the condition of 1% O_2_ (blue dots and line) in Figure 14a shows that the predicted results are in better agreement with the experimental values than the condition of 4% O_2_. When pressures are 2 and 5 atm in Figure 14b, the conclusion is essentially the same as for *p* = 4 atm.

**Figure 15:**
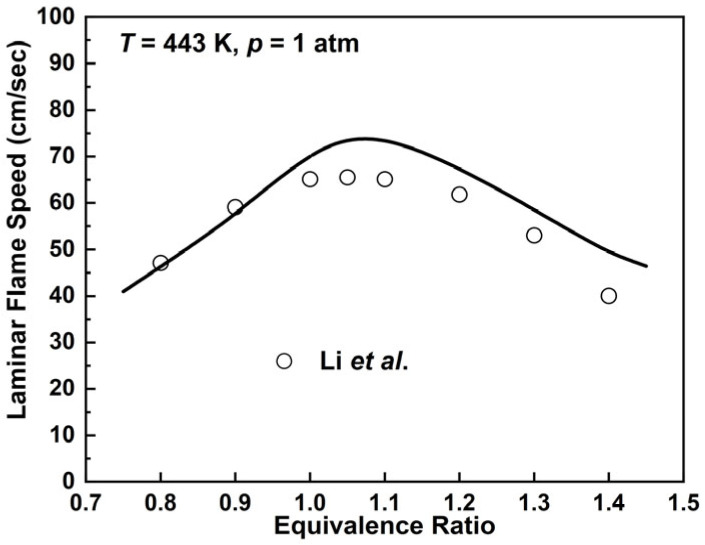
Laminar flame speeds for *n*-hexadecane/air mixtures at 1 atm, 443 K. Lines are mechanism prediction. Symbols are experimental data by Li et al. [57].

The predictions of laminar flame speed for the *n*-hexadecane mechanism are shown in Figure 15 and compared with experimental work by Li et al. [57] at the pressure of 1 atm and equivalent ratio range of 0.8–1.4. The *n*-hexadecane mechanism basically predicts the trend of the experimental values, which has an overprediction in the fuel-rich conditions while the deviation is relatively smaller in the fuel-lean combustion conditions. Based on the species and reactions of previously developed mechanisms, the results of the current *n*-hexadecane mechanism can be accepted with only four additional species as illustrated in Table 6.

### 2.3. Reaction Path Analysis

**Figure 16:**
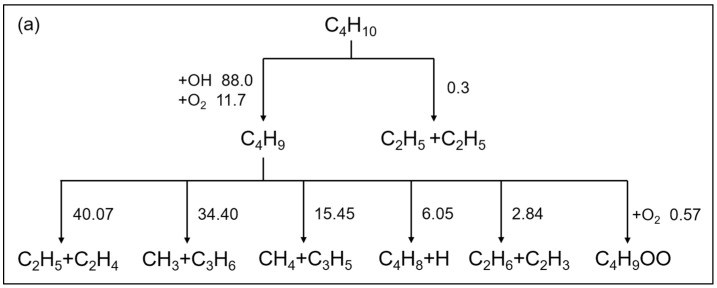

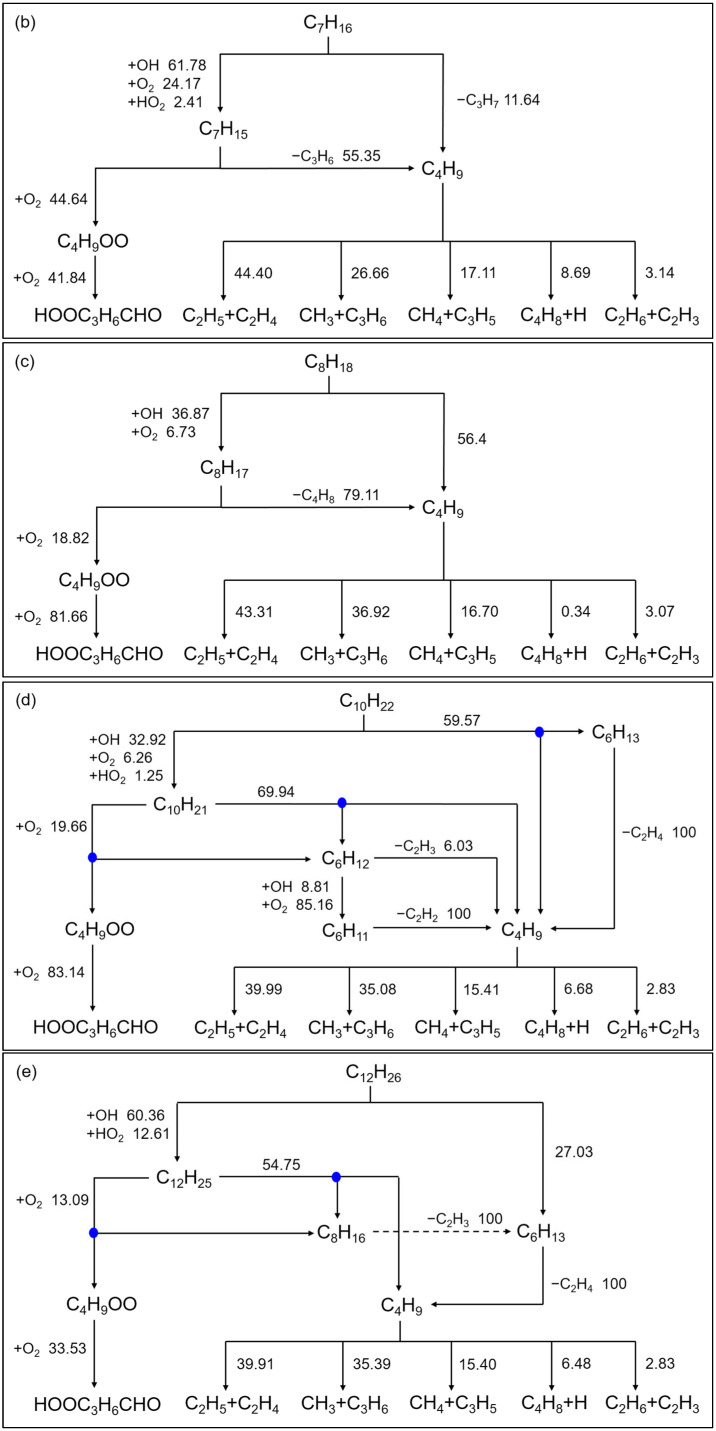

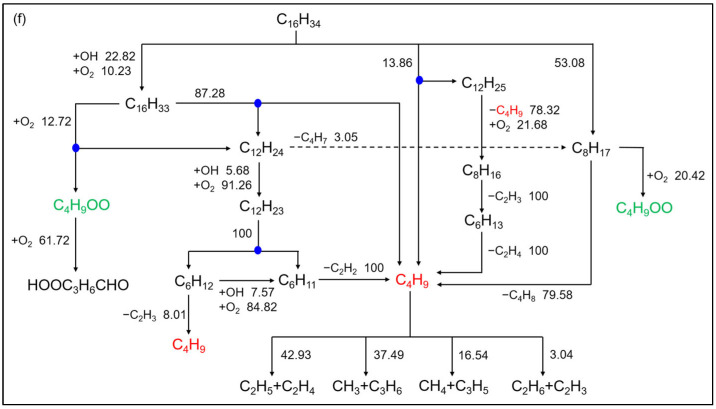
Reaction path analysis for stoichiometric mixture under 20 atm and 1100 K at ignition delay time of each mechanism correspondingly. (**a**) *n*-butane; (**b**) *n*-heptane; (**c**) *n*-octane; (**d**) *n*-decane; (**e**) *n*-dodecane; (**f**) *n*-hexadecane. Each number denotes the integral percentage contribution of the given path before ignition. The blue circle represents the path that separates the different products of the same reaction. In (**f**), C_4_H_9_ and C_4_H_9_OO are highlighted and the consumption paths of each species are shared independently.

In this section, reaction path analysis is performed for each mechanism developed in this work at *T* = 1100 K, *p* = 20 atm, *Φ* = 1, as shown in Figure 16. Combined with the reaction network, the compact mechanisms developed in this work are conducive to reaction path analysis.

In the *n*-butane mechanism, most C_4_H_10_ are consumed through oxidation reactions, with the proportion of H_2_O generated by OH hydrogen extraction reaction reaching 88%. The generated C_4_H_9_ are mainly consumed by different cracking reactions, with a higher proportion of C_2_H_5_+C_2_H_4_ and CH_3_+C_3_H_6_, which is also reflected in other mechanisms developed in this work.

In the *n*-heptane mechanism, a small portion of C_7_H_16_ is consumed through cracking reactions, while the majority is still consumed through oxidation reactions. Additionally, 44.64% of C_7_H_15_ are consumed by generating C_4_H_9_OO, while this proportion is very small in the *n*-butane mechanism. The remaining flux is contributed by the cracking reaction C_7_H_15_<=>C_3_H_6_+C_4_H_9_. The *n*-octane mechanism and *n*-heptane mechanism have similar reaction networks in structure, so their reaction path analysis results are also relatively similar. The difference is reflected in the proportion of each pathway, including the cracking reactions of fuels and the consumption of fuel radicals. The C_4_H_9_ cracking consumption between the *n*-octane mechanism and the *n*-heptane mechanism mainly differs in the generation of CH_3_+C_3_H_6_ and C_4_H_8_+H.

In the *n*-decane mechanism, due to the increase in intermediate species, the results for reaction path analysis are also more complex. The proportion of C_10_H_22_ consumed through oxidation and cracking is similar to that of the *n*-octane mechanism. Approximately 69.94% of C_10_H_21_ undergo a cracking process to generate C_6_H_12_ and C_4_H_9_, while 19.66% of C_10_H_21_ react with O_2_ to generate C_6_H_12_. The C_6_H_12_ mainly generate C_6_H_11_ through oxidation reactions. The C_6_H_13_ generated by the cracking of C_6_H_11_ and C_10_H_22_ are further converted 100% to C_4_H_9_.

In the *n*-dodecane mechanism, the amount consumed by C_12_H_26_ through cracking is less than half of the *n*-dodecane mechanism, and reactions with OH still dominate the oxidative depletion contribution of C_12_H_26_. For C_8_H_16_ generated by C_12_H_25_, they are basically consumed by cracking to generate C_6_H_13_. The reaction C_8_H_16_+OH<=>C_8_H_15_+H_2_O can be considered to have almost no contribution to C_8_H_16_ under current conditions. In the cracking paths of C_4_H_9_, the proportion of each group of products is almost the same as that of the *n*-decane mechanism.

In the reaction path analysis of the *n*-hexadecane mechanism, the network is more complex. For convenience, C_4_H_9_ and C_4_H_9_OO in some paths are displayed independently. The oxidation consumption of C_16_H_34_ is relatively low, about 32%. In the cracking process of C_16_H_34_, C_8_H_17_ contribute more than C_12_H_25_. C_8_H_17_ are mainly consumed by cracking to produce C_4_H_9_, rather than producing C_4_H_9_OO through oxidation. The consumption pathway of C_8_H_16_ generated through C_12_H_25_ is the same as in the *n*-dodecane mechanism. The main consumption pathway of C_16_H_33_ is through cracking to generate C_12_H_24_ and C_4_H_9_. The C_12_H_24_ further undergo an oxidation process to generate C_12_H_23_, which ultimately decompose. In the cracking reaction of C_4_H_9_, the path to generate C_4_H_8_+H is ignored.

### 2.4. CFD Calculation

**Figure 17:**
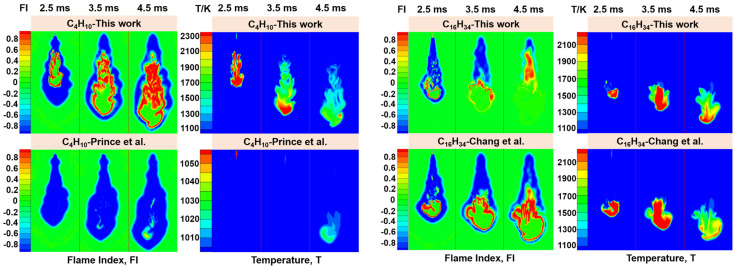
Distribution of flame index and temperature for C_4_ (Prince et al. [61]) and C_16_ (Chang et al. [20]) mechanisms.

Different combustion mechanisms were applied in the three-dimensional computational fluid dynamics (CFD) simulation. The settings of geometry model, spray model, combustion model and turbulence model in the simulation were consistent with the previous work by Wang et al. [58], in which the SAGE chemical solver [59] was adopted as the combustion model, and the LES approach [60] was adopted as the turbulence model. The mechanism for the smallest fuel molecule in this work, C_4_H_10_, was compared with the reduced mechanism proposed by Prince et al. [61] (47s-257r), and the mechanism for the largest fuel molecule, C_16_H_34_, was compared with the mechanism developed by Chang et al. [20] (40s-141r). The calculated distribution diagrams of flame index (FI) and temperature (T) are shown in Figure 17, in which 2.5 ms, 3.5 ms and 4.5 ms are shown. The FI can be used to observe the stability behaviors of different types of flames, where a positive FI indicates that premixed combustion is dominant and a negative FI means that diffusion combustion is dominant [58,62].

For the calculation results of C_4_H_10_, the FI for the mechanism developed in this work shows that a red inner layer is surrounded by a blue outer layer, indicating that the outer layer of the flame is mainly diffusion combustion, while the inner core of the flame is mainly premixed combustion. Furthermore, the position of the color region in the temperature distribution graph is basically consistent with the premixed flame region in the FI, which means that the contribution of flame temperature mainly comes from premixed combustion. However, the mechanism conducted by Prince et al. [61] is dominated by diffusion combustion in the FI graph under the current calculation setting and it does not present a significant temperature increase. Similarly, the reduced mechanism developed by Sharma et al. [63] does not yield satisfactory results either.

As for the calculation results of C_16_H_34_, the FI for the mechanism developed in this work is diffusion combustion enveloping premixed combustion at 2.5 ms, while at 3.5 ms, diffusion combustion is mainly distributed in the upstream of the flame and premixed combustion is mainly distributed in the downstream of the flame. At 4.5 ms, the premixed flame propagates upstream significantly. The high-temperature area in the temperature distribution transmits downwards continuously. The results of the mechanism proposed by Chang et al. [20] show that diffusion combustion envelops premixed combustion, and the position of the premixed flame region in the FI graph unifies with the color region in the temperature distribution.

## 3. Methods

In order to address the aforementioned issues of the existing mechanisms, the proposed minimized reaction network method for combustion kinetic mechanisms greatly reduces the number of chemical elementary reaction steps. This method compresses the variable space of the overall optimization of mechanisms and avoids the procedure of detailed mechanism reduction as this method is expected to be applied in turbulent combustion simulation directly.

**Figure 18:**
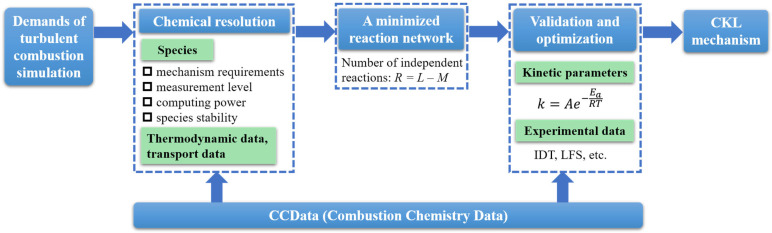
The main flowchart of modeling method.

The method is committed to controlling the number of species to match the demands of numerical simulation of turbulent combustion at the initiation of mechanism development as shown in Figure 18. Both CCData and CKL are sub-modules of the Combustion Dynamics platform of Sichuan University (CDS) [8], which is a specialized combustion chemistry database. CCData provides extensive data of fuel properties and serves as a valuable resource for supporting combustion kinetic studies. The CKL is a unified mechanism for multiple fuels, which contains combustion mechanisms developed in the minimized reaction network method, including the kinetic mechanisms developed in the present study and those of other typical fuels such as cycloalkanes and aromatic hydrocarbons, etc.

The single mechanism for each fuel is constructed hierarchically in a modular fashion in which the direction of all reactions is normalized in form, and the overall mechanism is ultimately formed by summarizing each single mechanism together. Therefore, the actual applications of the overall mechanism require an extraction process tailored to specific fuel requirements, as the overall mechanism may include redundant species and reactions. This implies that the mechanism for each fuel can be relatively independent and they can be combined with each other. Mechanism extraction is another distinctive feature, which serves as one of the assurances for achieving the goal of a unified mechanism for multiple fuels.

### 3.1. Chemical Resolution

The chemical resolution (expressed by the number of mechanism species *L*) of a combustion mechanism is firstly determined according to the computing capacity and resolution constraint on engineering applications of combustion numerical simulation.

Referring to the limitation of chemical resolution in most turbulent combustion flow fields, appropriate reaction species are selected to match the actual simulation requirements. When selecting species, factors such as specific requirements, measurement level, computational power and species stability should be taken into consideration. In order to reduce the number of chemical species, the highly active species of double radicals above C_2_ and the species that can hardly be characterized in the flow field measurements are excluded preferentially. The chemical resolution of the combustion mechanism for a single fuel is recommended to be controlled within 50. This resolution is sufficient to describe the general combustion kinetic properties, which can also be applied in the typical turbulent combustion models such as EDC and PDF when conducting the numerical simulation of turbulent combustion.

In practice, this method allows for the customized construction of mechanisms for a specific number of species. The mechanisms developed in this work are more generalized than we initially expected.

### 3.2. The Determination of Minimized Reaction Network

The single mechanism for each fuel is constructed hierarchically in a modular fashion in which the direction of all reactions is normalized in form, and the overall mechanism is ultimately formed by summarizing each single mechanism together. Therefore, the actual applications of the overall mechanism require the extraction process to be tailored to specific fuel requirements, as the overall mechanism may include redundant species and reactions. This implies that the mechanism for each fuel can be relatively independent and they can be combined with each other. Mechanism extraction is another distinctive feature, which serves as one of the assurances for achieving the goal of a unified mechanism for multiple fuels.

On the basis of the chemical resolution *L*, the independent reaction number *R* of the mechanism is further determined and the minimal reaction network is constructed subsequently.

For the combustion chemical process, all reactions reach simultaneous chemical equilibrium after a sufficient time from the initial state, thus all species are at equilibrium concentration in the system. The process of chemical reactions is the evolution of species from initial concentration to the final equilibrium. The driving force of this process is chemical potential and the task of combustion kinetics is to describe this process.

According to the chemical potential criterion, if a reaction reaches equilibrium, the chemical potential of each species meets:

(2) $$\underset{B}{\sum }A_{iB}\mu_{B}=0$$

where $A_{iB}$ is the stoichiometric coefficient, $\mu_{B}$ is the chemical potential. The chemical potential relationship of a mechanism can be written as homogeneous equations when all the reactions reach simultaneous equilibrium:

(3) Aμ=0

where **A** is the stoichiometric coefficient matrix of reactions, μ is the chemical potential column matrix. The rank *R**_A_*** of the matrix **A** obtained by elementary row transformation is the number of independent reactions of the mechanism. In fact, the independent reaction number can be obtained by the following relationship [64]:

*R**_A_*** = *L* − *M* (4)

where *M* is the rank of the atomic matrix. Generally, *M* is the number of actual atomic species involved in the reaction process of the combustion mechanism.

The minimal reaction network can be constructed since the independent reaction number *R**_A_*** of the mechanism is determined. The number of independent reactions determines the minimum number of elementary chemical reactions required to describe the chemical equilibrium state of the complex combustion reaction system. It is worth noting that independent reactions are not unique, and they are related to the selection of initial species. It is suggested to select the main fuels, O_2_ and H_2_ as the initial species for the combustion reaction system in which only C, H, O are involved, therefore other species can be obtained from combinations of these initial species.

If the evolution of species along with time in independent reactions under different conditions can be directly obtained, it is sufficient to describe the information of the combustion process. However, the evolution of species over time is generally difficult to acquire directly, therefore the law of mass action is frequently adopted and a list of elementary reactions with kinetic parameters is formed. Theoretically, the kinetic process of combustion can be described by the forward and reverse kinetic parameters of these independent reactions if rate constants are acquired. Since the independent reactions are not all elementary steps, there may be a lack of kinetic parameters. It is necessary to combine and replace these independent reactions with elementary reactions with known rate constants, which causes an appropriate increase in reaction step number.

For the sake of convenience, the actual construction process primarily focuses on establishing reaction connections among the selected species to ensure the formation of independent reactions. However, the selection of reactions sometimes requires careful consideration of various factors, which may result in the inclusion of multiple reactions and an appropriate increase in the number of reactions. 

### 3.3. Validation and Optimization

The kinetic parameters are determined through the overall optimization of the mechanism and the kinetic parameters are in the form of a classical two-parameter (*A*, *E_a_*) Arrhenius equation, in which the activation energy *E_a_* has clear physical significance.

The deficiencies of the modified three-parameter Arrhenius Equation (1) which is extensively adopted in the current combustion research have been interpreted [24]. In order to restore and ensure the physical meaning of the activation energy of *E_a_* as well as compress the variable space to facilitate the overall mechanism optimization, the classical two-parameter Arrhenius equation form is proposed to be adopted, that is,

(5) $$k=Ae^{-\frac{E_{a}}{RT}}$$

Such treatment can also avoid the problems caused by improper fitting of the three-parameter Arrhenius equation and assist us in visually assessing the rationality of parameters (*A*, *E_a_*) in engineering applications.

Ulteriorly, the kinetic parameters are optimized in a reasonable range to ensure the simulation accuracy of the mechanism as the contribution of multiple reaction channels is compressed in the minimized reaction network method. The final kinetic parameters of reactions are the result of two-parameterization, reaction path lumping and parameter optimization.

## 4. Conclusions

According to the demand of combustion mechanism resolution and the principle of simultaneous equilibrium of chemical reactions, the chemical potential relationship of a thermodynamic equilibrium system and the minimized reaction network with specified species were established. Subsequently, the minimized reaction network method of a complex reaction system was further proposed.

In this method, the number of chemical species and reaction steps can be extremely decreased, as the procedure of detailed mechanism reduction is avoided. In addition, the classical two-parameter (*A*, *E_a_*) form of the Arrhenius equation was suggested for the rate constants to guarantee the physical meaning of the activation energy for chemical reactions. Furthermore, the variable space of the mechanism can be compressed to reduce the difficulty of overall optimization. All reactions were adopted in the reversible form and there exists a cracking network for every considered fuel.

On the basis of the H_2_/C_1_-C_3_ mechanism which was also developed in the minimized reaction network method, the combustion mechanisms of typical *n*-alkanes developed in this work include 41 species and 83 reactions for *n*-butane, 42 species and 86 reactions for *n*-heptane, 42 species and 86 reactions for *n*-octane, 45 species and 91 reactions for *n*-decane, 46 species and 91 reactions for *n*-dodecane, 52 species and 104 reactions for *n*-hexadecane. We show that compact combustion mechanisms can be developed directly and reasonably by this method while sufficient prediction abilities are maintained, which brings convenience to engineering applications.

## Figures and Tables

**Table 1:** The reactions and kinetic parameters of the *n*-butane mechanism in this work.

Species	#	Reaction	*A* ^1^	*E_a_* ^1^
C_4_H_10_	R1	C_4_H_10_(+M)<=>2C_2_H_5_(+M)	1.52 × 10^16^	82,898
		Low pressure limit	4.72 × 10^18^	49,580
	R2	C_4_H_10_<=>C_4_H_9_+H	6.32 × 10^17^	90,000
	R3	C_4_H_10_+O_2_<=>C_4_H_9_+HO_2_	6.00 × 10^14^	45,340
	R4	C_4_H_10_+OH<=>C_4_H_9_+H_2_O	3.11 × 10^14^	3250
C_4_H_9_	R5	C_4_H_9_<=>CH_4_+C_3_H_5_	1.72 × 10^13^	28,900
	R6	C_4_H_9_<=>C_2_H_5_+C_2_H_4_	4.47 × 10^13^	28,900
	R7	C_4_H_9_<=>C_2_H_6_+C_2_H_3_	3.16 × 10^12^	28,900
	R8	C_4_H_9_<=>CH_3_+C_3_H_6_	4.19 × 10^13^	28,900
	R9	C_4_H_9_<=>H+C_4_H_8_	1.29 × 10^13^	28,900
	R10	C_4_H_9_+CH_4_<=>C_3_H_8_+C_2_H_5_	7.23 × 10^13^	28,900
	R11	C_4_H_9_+H<=>C_4_H_8_+H_2_	5.42 × 10^12^	0
	R12	C_4_H_9_+O_2_<=>C_4_H_9_OO	6.60 × 10^11^	1840
C_4_H_9_OO	R13	C_4_H_9_OO+O_2_<=>HOOC_3_H_6_CHO+OH	3.03 × 10^11^	1000
HOOC_3_H_6_CHO	R14	HOOC_3_H_6_CHO<=>OH+OC_3_H_6_CHO	1.05 × 10^13^	40,000
OC_3_H_6_CHO	R15	OC_3_H_6_CHO<=>CH_3_CHO+CH_3_CO	7.01 × 10^13^	1750
C_4_H_8_	R16	C_4_H_8_<=>C_3_H_5_+CH_3_	1.00 × 10^16^	72,900
	R17	C_4_H_8_<=>C_4_H_7_+H	6.32 × 10^16^	90,000
	R18	C_4_H_8_+O_2_<=>C_4_H_7_+HO_2_	2.00 × 10^15^	30,900
	R19	C_4_H_8_+OH<=>C_4_H_7_+H_2_O	3.52 × 10^14^	3970
C_4_H_7_	R20	C_4_H_7_<=>C_2_H_4_+C_2_H_3_	1.83 × 10^14^	27,200

^1^ Units: cm^3^, mol, s, cal, K.

**Table 2:** The reactions and kinetic parameters of the *n*-heptane mechanism in this work.

Species	#	Reaction	*A* ^1^	*E_a_* ^1^
C_7_H_16_	R21	C_7_H_16_<=>C_3_H_7_+C_4_H_9_	5.00 × 10^17^	77,000
	R22	C_7_H_16_<=>C_7_H_15_+H	6.32 × 10^16^	90,000
	R23	C_7_H_16_+O_2_<=>C_7_H_15_+HO_2_	4.20 × 10^13^	30,800
	R24	C_7_H_16_+OH<=>C_7_H_15_+H_2_O	1.28 × 10^14^	23,140
	R25	C_7_H_16_+HO_2_<=>C_7_H_15_+H_2_O_2_	5.36 × 10^15^	3880
C_7_H_15_	R26	C_7_H_15_<=>C_3_H_6_+C_4_H_9_	1.42 × 10^14^	30,000
	R27	C_7_H_15_+O_2_<=>C_3_H_6_+C_4_H_9_OO	9.08 × 10^12^	1840

^1^ Units: cm^3^, mol, s, cal, K.

**Table 3:** The reactions and kinetic parameters of the *n*-octane mechanism in this work.

Species	#	Reaction	*A* ^1^	*E_a_* ^1^
C_8_H_18_	R28	C_8_H_18_<=>2C_4_H_9_	2.50 × 10^18^	76,200
	R29	C_8_H_18_<=>C_8_H_17_+H	6.32 × 10^16^	90,000
	R30	C_8_H_18_+O_2_<=>C_8_H_17_+HO_2_	4.20 × 10^14^	30,800
	R31	C_8_H_18_+OH<=>C_8_H_17_+H_2_O	2.81 × 10^15^	2470
	R32	C_8_H_18_+HO_2_<=>C_8_H_17_+H_2_O_2_	1.28 × 10^14^	23,140
C_8_H_17_	R33	C_8_H_17_<=>C_4_H_8_+C_4_H_9_	1.42 × 10^14^	30,000
	R34	C_8_H_17_+O_2_<=>C_4_H_8_+C_4_H_9_OO	2.20 × 10^12^	1840

^1^ Units: cm^3^, mol, s, cal, K.

**Table 4:** The reactions and kinetic parameters of the *n*-decane mechanism in this work.

Species	#	Reaction	*A* ^1^	*E_a_* ^1^
C_10_H_22_	R35	C_10_H_22_<=>C_6_H_13_+C_4_H_9_	2.50 × 10^18^	76,200
	R36	C_10_H_22_<=>C_10_H_21_+H	6.32 × 10^17^	90,000
	R37	C_10_H_22_+O_2_<=>C_10_H_21_+HO_2_	4.20 × 10^13^	30,800
	R38	C_10_H_22_+OH<=>C_10_H_21_+H_2_O	5.36 × 10^14^	3880
	R39	C_10_H_22_+HO_2_<=>C_10_H_21_+H_2_O_2_	1.28 × 10^14^	23,140
C_10_H_21_	R40	C_10_H_21_<=>C_6_H_12_+C_4_H_9_	5.68 × 10^13^	30,000
	R41	C_10_H_21_+O_2_<=>C_6_H_12_+C_4_H_9_OO	1.10 × 10^12^	1840
C_6_H_13_	R42	C_6_H_13_<=>C_4_H_9_+C_2_H_4_	5.68 × 10^14^	30,000
C_6_H_12_	R43	C_6_H_12_+OH<=>C_6_H_11_+H_2_O	1.20 × 10^14^	4950
	R44	C_6_H_12_+O_2_<=>C_6_H_11_+HO_2_	2.00 × 10^14^	30,930
	R45	C_6_H_12_<=>C_4_H_9_+C_2_H_3_	1.89 × 10^14^	61,000
C_6_H_11_	R46	C_6_H_11_<=>C_4_H_9_+C_2_H_2_	7.12 × 10^15^	32,100

^1^ Units: cm^3^, mol, s, cal, K.

**Table 5:** The reactions and kinetic parameters of the *n*-dodecane mechanism in this work.

Species	#	Reaction	*A* ^1^	*E_a_* ^1^
C_12_H_26_	R47	C_12_H_26_<=>2C_6_H_13_	2.50 × 10^18^	76,200
	R48	C_12_H_26_<=>C_12_H_25_+H	6.32 × 10^16^	90,000
	R49	C_12_H_26_+O_2_<=>C_12_H_25_+HO_2_	4.20 × 10^15^	30,800
	R50	C_12_H_26_+OH<=>C_12_H_25_+H_2_O	5.36 × 10^16^	3880
	R51	C_12_H_26_+HO_2_<=>C_12_H_25_+H_2_O_2_	8.28 × 10^14^	20,140
C_12_H_25_	R52	C_12_H_25_<=>C_10_H_21_+C_2_H_4_	5.68 × 10^13^	25,000
	R53	C_12_H_25_+O_2_<=>C_8_H_16_+C_4_H_9_OO	9.08 × 10^12^	1840
C_8_H_16_	R54	C_8_H_16_<=>C_6_H_13_+C_2_H_3_	1.89 × 10^16^	61,000
	R55	C_8_H_16_+OH<=>C_8_H_15_+H_2_O	1.20 × 10^13^	4950
C_8_H_15_	R56	C_8_H_15_<=>C_5_H_9_+C_3_H_6_	2.11 × 10^15^	50,310
C_5_H_9_	R57	C_5_H_9_<=>C_2_H_4_+C_3_H_5_	2.11 × 10^15^	20,310

^1^ Units: cm^3^, mol, s, cal, K.

**Table 6:** The reactions and kinetic parameters of the *n*-hexadecane mechanism in this work.

Species	#	Reaction	*A* ^1^	*E_a_* ^1^
C_16_H_34_	R58	C_16_H_34_<=>2C_8_H_17_	2.50 × 10^19^	76,200
	R59	C_16_H_34_<=>C_4_H_9_+C_12_H_25_	2.50 × 10^18^	76,200
	R60	C_16_H_34_<=>C_16_H_33_+H	1.58 × 10^16^	97,510
	R61	C_16_H_34_+O_2_<=>C_16_H_33_+HO_2_	4.20 × 10^13^	30,800
	R62	C_16_H_34_+OH<=>C_16_H_33_+H_2_O	5.36 × 10^14^	3880
	R63	C_16_H_34_+HO_2_<=>H_2_O_2_+C_16_H_33_	1.28 × 10^14^	23,140
C_16_H_33_	R64	C_16_H_33_<=>C_4_H_9_+C_12_H_24_	8.42 × 10^14^	30,000
	R65	C_16_H_33_+O_2_<=>C_4_H_9_OO+C_12_H_24_	7.08 × 10^12^	1840
C_12_H_24_	R66	C_12_H_24_<=>C_4_H_7_+C_8_H_17_	1.89 × 10^14^	61,000
	R67	C_12_H_24_+O_2_<=>C_12_H_23_+HO_2_	2.00 × 10^14^	30,930
	R68	C_12_H_24_+OH<=>C_12_H_23_+H_2_O	1.20 × 10^14^	4950
C_12_H_23_	R69	C_12_H_23_<=>C_6_H_11_+C_6_H_12_	7.12 × 10^15^	32,100

^1^ Units: cm^3^, mol, s, cal, K.

## Data Availability

Data are contained within the article.

## Supplementary Materials

The following supporting information can be downloaded at: https://www.mdpi.com/article/10.3390/molecules28237695/s1, The kinetic mechanism for each fuel, therm.dat and trans.dat.
